# Variations of bacterial community during the decomposition of *Microcystis* under different temperatures and biomass

**DOI:** 10.1186/s12866-019-1585-5

**Published:** 2019-09-04

**Authors:** Shuren Wang, Dayong Zhao, Jin Zeng, Huimin Xu, Rui Huang, Congcong Jiao, Lin Guo

**Affiliations:** 1 State Key Laboratory of Hydrology-Water Resources and Hydraulic Engineering, Joint International Research Laboratory of Global Change and Water Cycle, Hohai University, Xikang Road 1, Nanjing, 210098 China; 2 State Key Laboratory of Lake Science and Environment, Nanjing Institute of Geography and Limnology, Chinese Academy of Sciences, 73 East Beijing Road, Nanjing, 210008 China; 3 Department of Biological and Environmental Sciences, Texas A&M University, Commerce, TX 76129 USA

**Keywords:** *Microcystis* decomposition, Biomass, Temperature, Bacterial community

## Abstract

**Background:**

The decomposition of *Microcystis* can dramatically change the physicochemical properties of freshwater ecosystems. Bacteria play an important role in decomposing organic matters and accelerating the cycling of materials within freshwater lakes. However, actions of the bacterial community are greatly influenced by temperature and the amount of organic matter available to decompose during a bloom. Therefore, it is vital to understand how different temperatures and biomass levels affect the bacterial community during the decomposition of *Microcystis*.

**Results:**

*Microcystis* addition reduced the diversity of bacterial community. The composition of bacterial community differed markedly between samples with different biomass of *Microcystis* (no addition, low biomass addition: 0.17 g/L, and high biomass addition: 0.33 g/L). In contrast, temperature factor did not contribute much to the different bacterial community composition. Total nitrogen (TN), total phosphorus (TP), total organic carbon (TOC), ammonia nitrogen (NH_4_^+^-N) and oxidation-reduction potential (ORP) were the key measured environmental variables shaping the composition of bacterial community.

**Conclusions:**

Decomposition of *Microcystis* changed the physicochemical characteristics of the water and controlled the diversity and composition of the bacterial community. *Microcystis* biomass rather than temperature was the dominant factor affecting the diversity and composition of the bacterial community.

**Electronic supplementary material:**

The online version of this article (10.1186/s12866-019-1585-5) contains supplementary material, which is available to authorized users.

## Background

With increased anthropogenic pressure within watersheds, eutrophication has become one of the most serious environmental problems facing Chinese freshwater lakes [1]. *Microcystis* blooms, affiliated to Cyanobacteria, are common in eutrophic freshwater lakes and harmful to human health [2, 3]. Bacteria are a key component of aquatic ecosystems and play important roles in decomposing of organic matters derived from phytoplankton [4, 5]. Bacterial communities associated with the growth of bloom-forming freshwater phytoplankton have received considerable attentions [5, 6]. However, relatively few studies have assessed the response of freshwater bacterial communities to the decomposition of *Microcystis*. The bacteria-*Microcystis* relationship has important implications in aquatic ecosystems.

Previous studies have reported obvious changes in environmental variables as well as bacterial communities due to the decomposition of *Microcystis* [7–9]. The release of dissolved organic matter (DOM) by bloom-forming *Microcystis* is a ubiquitous process in eutrophic freshwater lakes [10]. The rapid depletion of dissolved oxygen was always associated with bloom decomposition [11]. Li et al. [7] observed spatial and temporal heterogeneity in the composition of both planktonic and particle-attached bacterial communities in an anoxic zone of Lake Taihu, the anoxia being caused by *Microcystis* decomposition. Zhao et al. [8] constructed microcosms to characterize the bacterial community composition in water and sediment during *Microcystis* decomposition and found that marked changes of dissolved organic carbon (DOC) and pH might be responsible for variations in bacterial communities. Temperature is an important factor driving the growth and metabolism of microorganisms. Xing et al. [9] simulated the degradation of *Microcystis* in anoxic water columns and found that temperature had a great influence on the bacterial community composition. Therefore, both temperature and *Microcystis* biomass are key factors influencing the microbial communities during *Microcystis* degradation.

Here, we took both *Microcystis* biomass and temperature into account to understand how the *Microcystis* decomposition affected the bacterial community. We constructed microcosms containing different amounts of *Microcystis* biomass and incubated under different temperatures. Water samples were collected at the time point of intense *Microcystis* decomposition. Water characteristics were examined and the 454 pyrosequencing was used to investigate the diversity and composition of the bacterial community in water samples. Our study aimed to (1) understand how *Microcystis* decomposition affected the diversity and composition of bacterial community; (2) determine whether temperature or *Microcystis* biomass was the dominant factor controlling freshwater bacterial communities during *Microcystis* decomposition.

## Results

### Environmental variables during the decomposition of *Microcystis*

**Fig. 1:**
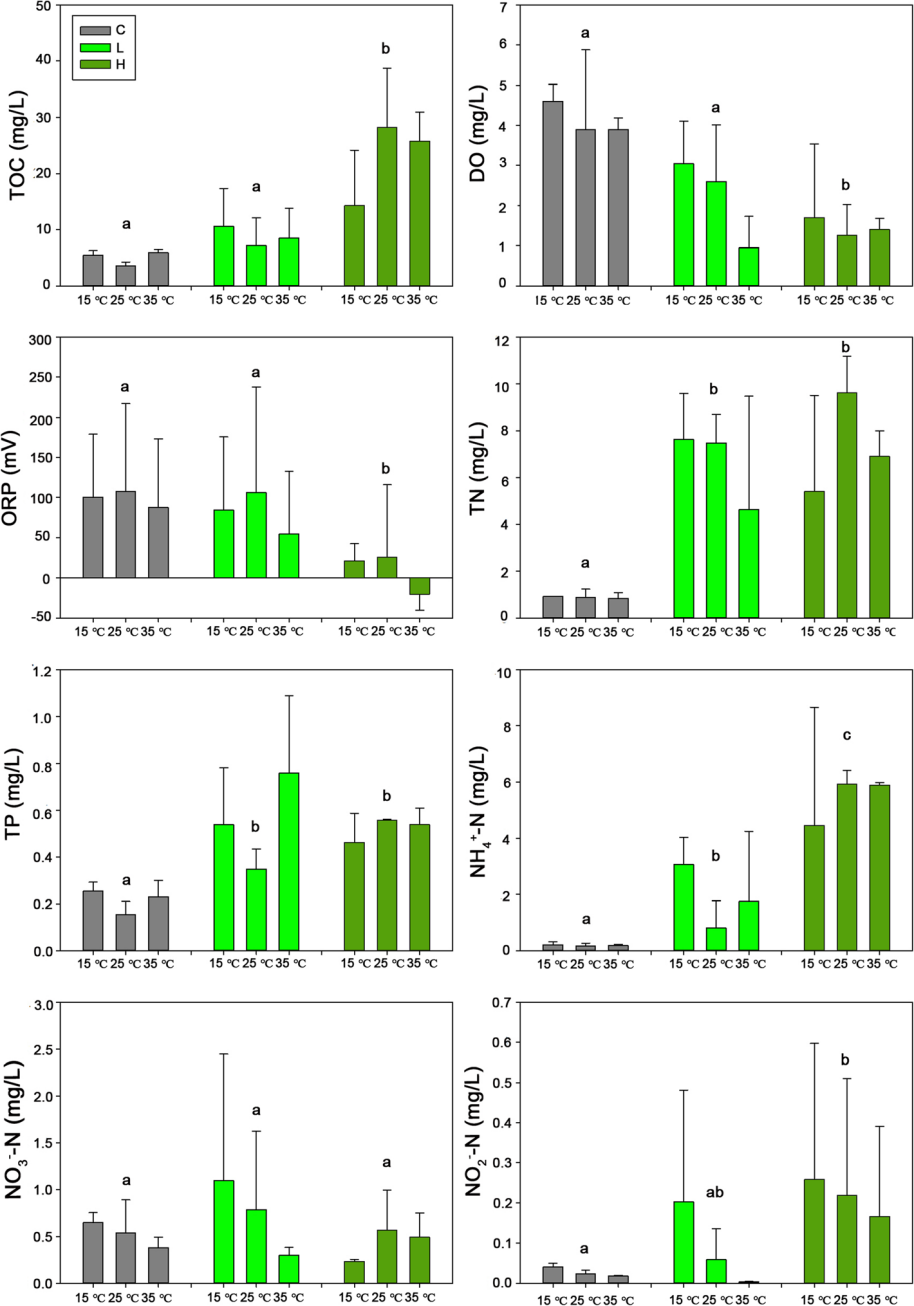
Environmental variables of different *Microcystis* addition treatments. TOC, total organic carbon; DO, dissolved oxygen; ORP, oxidation-reduction potential; TN, total nitrogen; TP, total phosphorus; NH_4_^+^-N, ammonia nitrogen; NO_3_^−^-N, nitrate nitrogen; NO_2_^−^-N, nitrite nitrogen. C, without addition of *Microcystis*; L, low *Microcystis* biomass treatment; H, high *Microcystis* biomass treatment. Different lowercase letters indicate significant difference (Duncan’s multiple range test, *P* < 0.05)

Physicochemical properties in water samples of different treatments were shown in Fig. 1. Marked changes were observed for the treatments with highest *Microcystis* biomass addition (H treatment). Overall, significant higher concentrations of TOC, TN, TP, NH_4_^+^-N and NO_2_^−^-N were observed in the treatments with *Microcystis* addition (H and L treatments) compared with the control treatment (without *Microcystis* addition). The DO and ORP decreased significantly in high *Microcystis* biomass addition (H) treatment. In contrast, little difference was found for the measured environmental variables between different temperature treatments (15 °C, 25 °C, 35 °C) (Additional file 1: Figure S1).

### Diversity and composition of the bacterial community within the different treatments

**Fig. 2:**
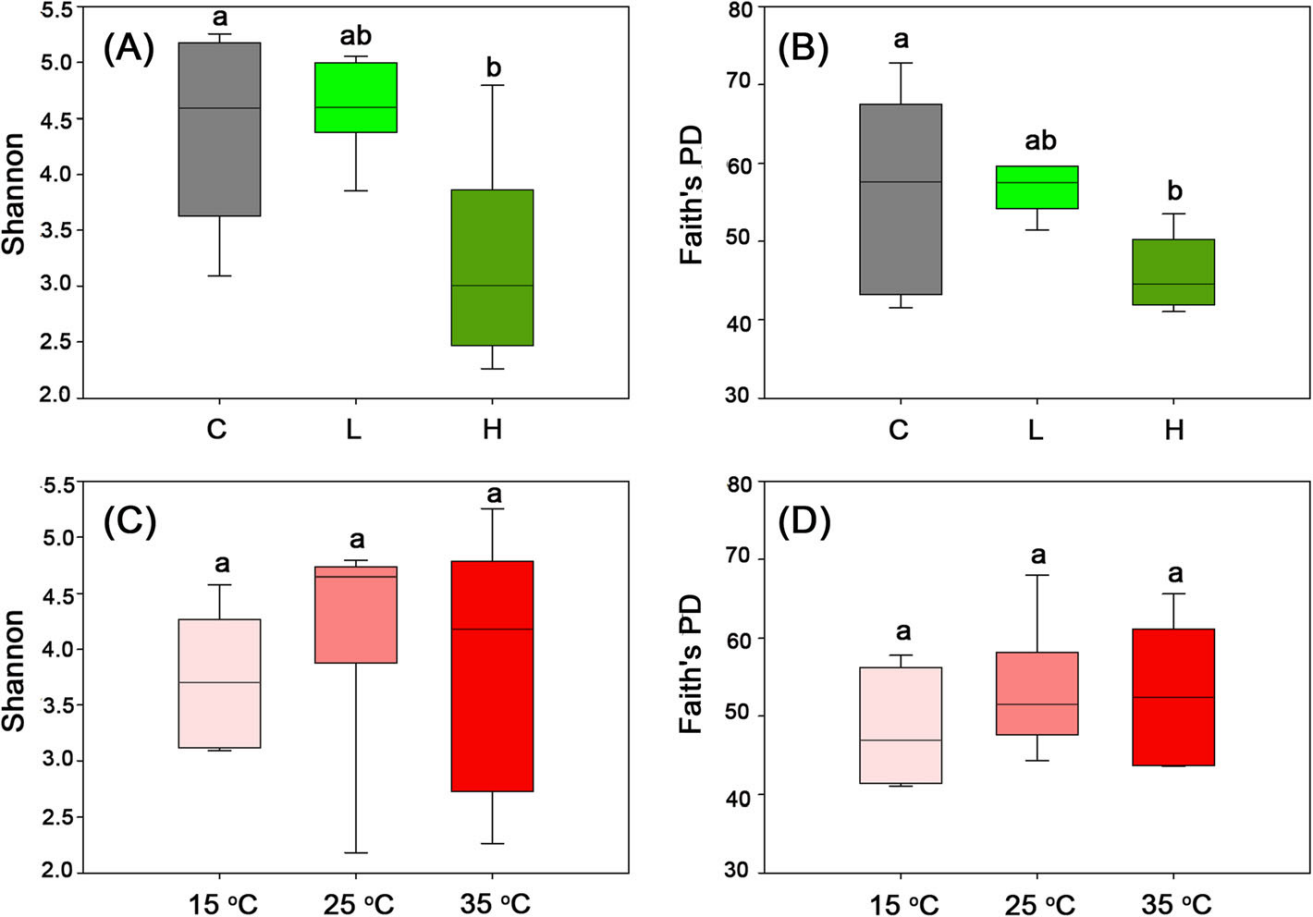
Alpha diversity of bacterial communities from different treatments. **a**, **b**: groups divided by *Microcystis* biomass; **c**, **d**: groups divided by temperature. Shannon, Shannon-Weiner index; Faith’s PD, the Faith’s phylogenetic diversity. C, without addition of *Microcystis*; L, low *Microcystis* biomass treatment; H, High *Microcystis* biomass treatment. Different lowercase letters indicate significant difference (Duncan’s multiple range test, *P* < 0.05)

Two different diversity indices (Shannon-Wiener index and Faith’s PD) were used to quantify the diversity of bacterial community of different treatments (C, L, H; 15 °C, 25 °C, 35 °C). Both indices showed the same pattern. In Fig. 2a and b, significant decrease in the bacterial community of H treatment was found (*P* < 0.05), whereas no significant difference was observed in the bacterial community of the C and L treatments. In Fig. 2c and d, diversity of bacterial community did not differ significantly for different temperature treatments (*P* > 0.05).

**Fig. 3:**
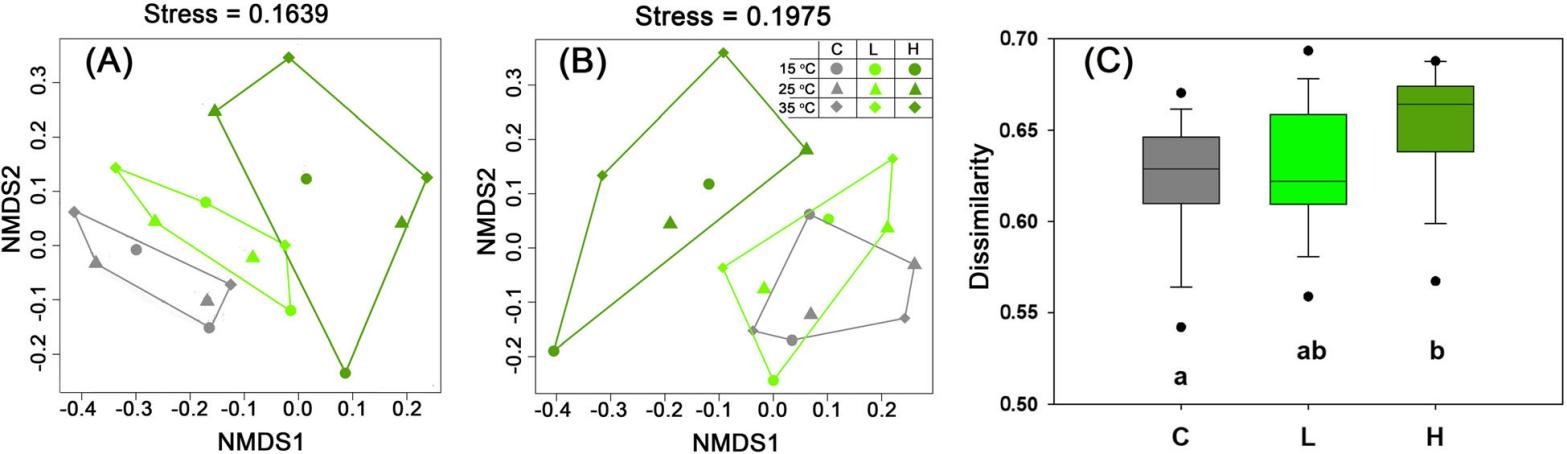
Dissimilarity of bacterial communities based on phylogenetic distance. **a**, **c**: based on Bray-Curtis distance; **b**: based on unweighted UniFrac distance. C, without *Microcystis* addition; L, low *Microcystis* biomass treatment; H, High *Microcystis* biomass treatment. Different lowercase letters indicate significant differences (Duncan’s multiple range test, *P* < 0.05)

Based on the Bray-Curtis and unweighted UniFrac distance, non-metric multidimensional scaling (NMDS) was performed to characterize the dissimilarity of bacterial community composition of the water samples. The bacterial community composition of the C, L and H treatments were grouped into three distinct clusters based on the Bray-Curtis distance (Fig. 3a). However, the bacterial community composition of the C and L groups was not clearly separated based on the unweighted UniFrac distance (Fig. 3b). The dissimilarity of the bacterial community composition of the H treatment group (0.66 ± 0.03) was significant higher than those of the C (0.62 ± 0.03) and L (0.62 ± 0.02) treatment groups (*P* < 0.05) (Fig. 3c). The results of permutational multivariate analysis of variance (PERMANOVA) showed significant differences in the composition of the bacterial communities of the C and H (*P* < 0.01) as well as the L and H groups (*P* < 0.01) (Table 1). In contrast, temperature only maintained little effects on controlling the bacterial community composition (Table 1).

**Table 1:** Results of permutational multivariate analysis of variance (PERMANOVA) between different treatment groups

Groups 1	C	L	H
C	–	*P* = 0.245	*P* = 0.003**
L	*R*^*2*^ = 0.099	–	*P =* 0.005**
H	*R*^*2*^ = 0.198	*R*^*2*^ = 0.170	–
Groups 2	15 °C	25 °C	35 °C
15 °C	–	*P* = 0.512	*P* = 0.185
25 °C	*R*^*2*^ = 0.088	–	*P* = 0.862
35 °C	*R*^*2*^ = 0.106	*R*^*2*^ = 0.066	–

Groups 1: groups divided by different *Microcystis* biomass; Groups 2: groups divided by different temperature. C, control treatment without addition of *Microcystis*; L, Low *Microcystis* biomass treatment; H, High *Microcystis* biomass treatment. **P < 0.01

### Taxonomic features of bacterial communities during *Microcystis* decomposition

**Fig. 4:**
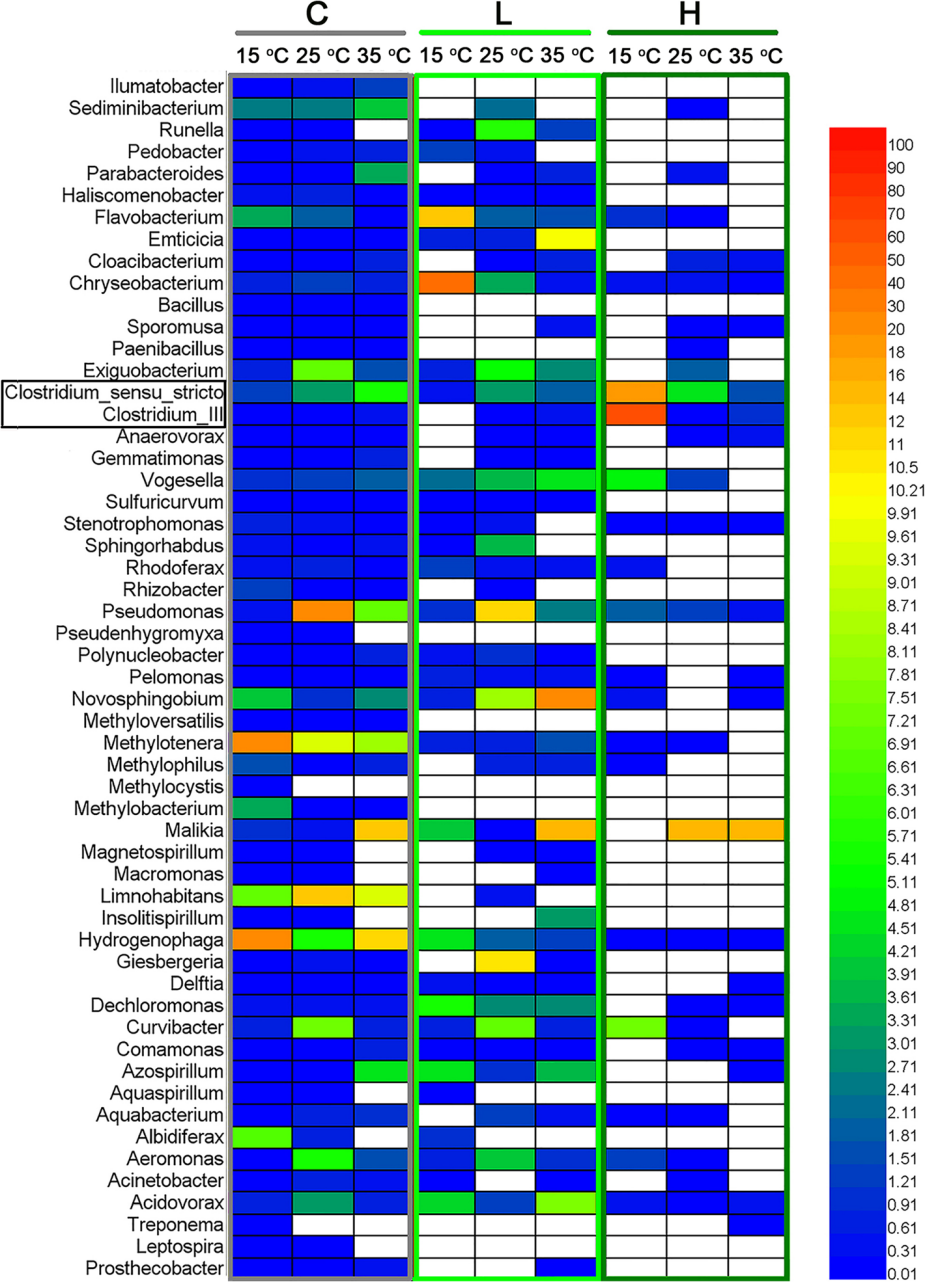
Relative abundance of the top 10 genus in different treatment groups. C, without addition of *Microcystis*; L, low *Microcystis* biomass; H, high *Microcystis* biomass. The color from blue to red indicates average relative abundance of genus in each treatment groups

The relative abundance at the phylum level calculated from the obtained bacterial sequences was presented in Additional file 2: Table S1. On average, Betaproteobacteria (52.98%), Bacteroidetes (10.96%), and Gammaproteobacteria (9.34%) were the dominant phyla, followed by Alphaproteobacteria (7.50%), Firmicutes (7.23%), Actinobacteria (5.51%), Planctomycetes (1.36%), Parcubacteria (1.27%) and Verrucomicrobia (1.09%). Other phyla including Fusobacteria and Chloroflexi were found at low abundance (< 1%). Variations of the six dominant phyla were tested against different temperatures or *Microcystis* biomass additions (Additional file 3: Figure S2). The relative abundance of Firmicutes increased significantly with the addition of *Microcystis* biomass, however, decreased significantly with the elevated temperature (*P <* 0.05). Actinobacteria showed an opposite pattern. Variations of the relative abundance of the dominant phyla can be found in Additional file 4: Figure S3). The relative abundance of the top ten dominant genera in different treatments was shown in Fig. 4. The number of bacterial genera in the *Microcystis*-added treatments (especially the H group) was remarkably lower than that of the C group (Fig. 4). Several genera were depleted during the decomposition of *Microcystis*. However, temperature did not show any consistent effect on the number of bacterial genera among different biomass treatments.

### Relationships between environmental variables and bacterial community composition

Mantel test was used to investigate the relationships between bacterial communities and the measured environmental variables. Both OTU-based Bray-Curtis distance and phylogenetic tree-based unweighted UniFrac distance were taken into account. NH_4_^+^-N (*P* < 0.001), TOC (*P* < 0.001), ORP (*P* < 0.001) and TN (*P* < 0.05) were significantly correlated with both of the bacterial community distance matrices (Table 2).

**Table 2:** Correlations as determined by Mantel tests between the bacterial community composition (Bray-Curtis and the unweighted UniFrac dissimilarity) and the measured environmental variables

Mantel test R	Bray-Curtis distance	UniFrac distance
NH_4_^+^-N	0.4025***	0.3844**
TOC	0.3329***	0.4991***
ORP	0.3307***	0.5004***
TN	0.2233*	0.1687*
TP	0.0814	0.0327
DO	0.1781	0.0777
NO_2_^−^-N	0.0778	−0.1874
NO_3_^−^-N	0.0154	−0.1101

*NH*_*4*_^*+*^*-N* Ammonia nitrogen, *TOC* Total organic carbon, *ORP* Oxidation-reduction potential, *TN* Total nitrogen, *TP* Total phosphorus, *DO* Dissolved oxygen, *NO*_*2*_^*—*^*N* Nitrite nitrogen, *NO*_*3*_^*—*^*N* Nitrate nitrogen. *P < 0.05, **P < 0.01, ***P < 0.001

In order to increase the robustness of the results, canonical correspondence analysis (CCA) was employed (Additional file 5: Figure S4). The two dominant axis totally explain 31.9% species-environment correlation. Community based on taxa abundance showed clustering characteristics with different *Microcystis* addition. TOC, DO and NH_4_^+^-N were the main environmental factors driving the community differentiation. According to the results of forward selection and ordination (Additional file 6: Table S2), ORP (*P* < 0.001), NH_4_^+^-N (*P* < 0.01) and TOC (*P* < 0.05) were the most significant environmental factors driving the bacterial community composition.

## Discussion

### Decomposition of *Microcystis* modifies the diversity of bacterial community

Remarkable decreases in the diversity of bacterial community were observed for the *Microcystis*-addition treatments after 5-day incubation. The lowest diversity of bacterial community was observed in the H group (Fig. 2). Wang et al. [12] investigated the diversity of the bacterial community in nonylphenol-degrading sediments and found that the Shannon-Wiener index decreased with incubation time. The decomposition of *Microcystis* also reduced the diversity of bacterial community. However, Dilly et al. [13], investigating the bacterial community diversity in relation to the decomposition of crop residues, found a pattern of increased bacterial diversity with decomposition. The decreased diversity of our results in the H group was mainly due to the harsh environments (Low DO and ORP conditions) that favored the growth of specific bacterial groups [7], which could be a process of environmental filtering. The depletion of several bacterial genera in *Microcystis*-added groups in comparison with the control treatments also confirmed these results (Fig. 4).

Zhou et al. [14] indicated that nutrient additions could potentially weaken competition among different bacterial species and stimulate an increase in population growth. However, remarkable decreases in bacterial community diversity were observed in our experiments. The contradictory results could be attributed to the differences in the initial states of the two studies. The geochemical conditions in Zhou et al.’s study were relatively toxic to many organisms (low pH and high concentrations of nitrate, aluminum and other metals) and had a limited carbon source. However, the initial state of our study was not a harsh environment. In the present study, water was collected from a eutrophic lake (TN, 0.86 mg/L; TP, 0.22 μg/L; TOC, 5.92 mg/L; DO, 6.4 mg/L; pH, 7.44; NH_4_^+^-N, 0.2 mg/L; ORP, 51 mv). Therefore, the addition of *Microcystis* did not stimulate the growth of a variety of bacterial groups. However, the decomposition of large amounts of *Microcystis* biomass represented an extreme perturbation for the bacterial community. The consumption of the dissolved oxygen during decomposition produced an environmental stress for the bacterial community [11]. Moreover, toxins produced by *Microcystis* were released to the waters during its decomposition [15].

### Effects of *Microcystis* biomass and temperature on the bacterial community composition during the decomposition of *Microcystis*

The clear separation among the C, L and H groups (Fig. 3a) indicated that the addition of *Microcystis* affected the composition of the bacterial community (Fig. 3a). Meanwhile, higher dissimilarity of bacterial community was also observed in the H group (Fig. 3c). The scatter of the H group could be resulted from a divergence of species drove by the violent *Microcystis* decomposition compared with the other two groups. The pattern of phylogenetic-based beta diversity of bacterial community also highlighted the great effects of *Microcystis* decomposition of high biomass (Fig. 3b). Previous studies have verified the dynamic changes of bacterial community composition during *Microcystis* decomposition in freshwater lakes [16, 17]. Li et al. [7] reported strong relationships between the bacterial community composition and *Microcystis* decomposition, which were apparent along a gradient of *Microcystis* biomass. In lake sediments, the addition of *Microcystis* biomass (both 200 μg/L and 2000 μg/L) also induced marked shifts in the composition of the bacterial community [16]. Here, we found the biomass of added *Microcystis* played more important roles than temperature in altering the composition of bacterial communities during the decomposition of *Microcystis*.

Some studies showed that bacterial community composition was related to the availability of organic matter, dissolved oxygen and pH [7, 18]. In our study, the rapid decay of *Microcystis* created an environment with a higher concentration of TN, TP, TOC and lower ORP and DO (Fig. 1). The results of the Mantel test demonstrated that TOC (*P* < 0.001), ORP (*P* < 0.001), NH_4_^+^-N (*P* < 0.001) and TN (*P* < 0.05) were significant environmental variables in correlation with bacterial community composition (Table 2). Meanwhile, the result of CCA indicated that TOC, NH_4_^+^-N and DO were the key environmental factors driving the bacterial community composition (Additional file 5: Figure S4 and Additional file 6: Table S2). Previous studies have also found the correlations between DOC [19], TN [20], NH_4_^+^-N [20] and the composition or diversity of bacterial community.

Gucht et al. [21] found that Proteobacteria was the dominant phylum in freshwater ecosystems, the relative abundance of this phylum being very high in our samples, especially for Betaproteobacteria (Additional file 4: Figure S3)*.* The relative abundance of Firmicutes increased significantly after the addition of *Microcystis* compared with the C group (Additional file 3: Figure S2). The genus *Clostridium* affiliated with Firmicutes was influenced strongly by the decomposition of *Microcystis* (Fig. 4). Previous study has observed that *Clostridium* was the dominant group (accounting for 72% of the sequenced clones) during *Microcystis* blooms [9]. Moreover, Zhao et al. [8] also found an increased abundance of *Clostridium* during the anaerobic decomposition of organic matter. Actinobacteria play an important role in the material cycle of freshwater ecosystems [22]. Although the decomposition of *Microcystis* provided a large amount of TOC, TN and TP, the process consumed much dissolved oxygen in the microcosm, which could partly explain the decreased relative abundance of some aerobic bacteria, such as Actinobacteria [23].

## Conclusions

This study demonstrated that the biomass of added *Microcystis* was a key variable that shaped the composition and diversity of bacterial community in the microcosms, whereas temperature played little roles in driving the bacterial community composition. The intense decomposition of higher biomass of *Microcystis* reduced bacterial diversity. *Clostridium* affiliated to Firmicutes increased markedly during the process. TN, TP, TOC, NH_4_^+^-N and ORP were the dominant environmental factors correlated with bacterial community composition. The findings of the present study would be helpful for further understanding the impacts of cyanobacterial blooms on freshwater ecosystems.

## Methods

### Experimental design

Microcosms were built using sediments, lake water and *Microcystis* assemblages collected from Meiliang Bay (N 31^o^29’14″, E 120^o^12’41″) of Lake Taihu, China, a large shallow eutrophic freshwater lake that suffering from serious *Microcystis* blooms every year in summer. Before microcosms constructed, the sediments were sieved (1-mm mesh) to remove macrofauna and large particles. Lake water was filtered with a plankton net to collect *Microcystis* colonies. The *Microcystis* colonies were then rinsed three times with ultrapure water before use. The *Microcystis* spp. constituted up to 97% of the total phytoplankton cells by using microscopic examination. The collected *Microcystis* colonies were frozen at − 80 °C and then freeze-dried. The microcosms were built using plexiglas columns (diameter: 16 cm; height: 30 cm). The homogenized sediments and water were transferred into the columns and stabilize for 2 days under the designated temperature in an incubator. The results of our experiments indicated that the sterilization of *Microcystis* before biomass addition would not significantly affect the diversity and composition of bacterial community in water of the microcosms (Additional file 7: Methods and results of the supplementary experiments; Additional file 8: Figure S5; Additional file 9: Figure S6 and Additional file 10: Figure S7). The freeze-dried *Microcystis* colonies were added to the microcosms. Based on the *Microcystis* biomass in Lake Taihu reported in previous investigations [7, 8], three different *Microcystis* biomass treatments were designed: (1) the H treatment with a high *Microcystis* biomass addition (**H**, 0.33 g/L), (2) the L treatment with a low *Microcystis* biomass addition (**L**, 0.17 g/L) and (3) the control treatment without *Microcystis* addition (**C**, 0 g/L). The microcosms were incubated under three different temperatures, 15, 25 and 35 °C, to investigate the effects of temperature on the bacterial communities during decomposition process.

### Sampling and physicochemical analysis

Referring to the results of previous studies [7, 8], marked variations in water properties were expected on days 2 to 5 after the addition of *Microcystis*, which signified violent decomposition. Therefore, samples were collected on day 5 for microbial community analysis. Water characteristics, including pH, oxidation-reduction potential (ORP) and dissolved oxygen (DO) were analyzed with electrodes during incubation. Total nitrogen (TN) and total phosphorus (TP) were determined following Jin et al. [24]. The concentrations of ammonia nitrogen (NH_4_^+^-N), nitrate nitrogen (NO_3_^−^-N), nitrite nitrogen (NO_2_^−^-N) were measured by a continuous flow analyzer (San++, SKALAR, Netherlands) using the water samples collected after they passed through a 0.45 μm pore-size filter. Bacteria were collected with a 0.22 μm pore-size polycarbonate membrane filter (Millipore, Billerica, MA, USA). The filters were stored at − 80 °C before further processing [25].

### DNA extraction, PCR and pyrosequencing

We extracted bacterial genome DNA from each water sample using the E.Z.N.A.® Water DNA Kit (Omega Biotek, Doraville, GA) [26]. A nano-Drop ND-1000 spectrophotometer (NanoDrop Technologies, Wilmington, DE, USA) verified both the quality and quantity of the extracted DNA, and the extracted DNA was then amplified by PCR. The primers 533R (TTACCGCGGCTGCTGGCAC) and 8F (AGAGTTTGATCCTGGCTCAG), fixed with the Roche 454 sequencing adapters, were used to amplify the bacterial 16S rRNA genes. Meanwhile, individual 10-bp barcode sequences of each sample were linked to the reverse primer 533R [27]. To reduce any error resulting from PCR amplification, three parallels were employed for each PCR reaction. The total volume of the PCR mixture was 20 μL, including 4 μL 5× Prime STAR Buffer (plus Mg^2+^), 0.4 μL FastPfu DNA polymerase (2.5 U/ μL), 0.4 μL for each of the forward and reverse primers (5 μM), 2 μL dNTPs (2.5 mM), 10 ng DNA template (about 10 ng/μL). The thermal cycling conditions for PCR amplification followed the methods of Schloss et al. [28]. After being pooled, the PCR products were verified by gel electrophoresis, then purified using the AxyPrep DNA gel purification kit (Axygen Biotechnology Hangzhou Ltd., Hangzhou, China). After quantifying the purified PCR products, > 200 ng DNA of each sample was sent to the Majorbio Company in Shanghai for pyrosequencing on a 454 FLX Titanium platform (Roche).

### Data processing and statistical analysis

The DNA sequence data were trimmed and de-noised following the online 454 standard operating procedure (SOP) of the Mothur 1.39.0 software package [28, 29]. Sequences that were not fully matched with primers, having an average quality score < 27 or containing any ambiguous reads, homopolymers > 8 nt or sequences < 200 bp (excluding the barcode or primer) were removed from further analysis [30]. The remaining sequences were converted by reverse complementation and aligned against a bacterial SILV16S rRNA gene template (using NAST algorithm) [28]. Putative chimeric sequences and sequencing errors were removed by using the *chimera.uchime* and *pre.cluster* commands in Mothur [31]. The sequences were classified using a Bayesian classifier referring to the 16S rRNA gene training set of Ribosomal Database Project (http://rdp.cme.msu.edu) with the threshold of 80% [32]. Then, unknown sequences and sequences affiliated with Archaea, Cyanobacteria, chloroplast were excluded from further analysis.

Operational taxonomic units (OTUs) were clustered within the remaining sequences at a 3% dissimilarity [33]. The same number of sequences was drawn randomly from each dataset for estimating diversity at the same sequencing depth. The OTU richness was calculated by Shannon index (*H*) according to the OTU table, while the phylogenetic diversity (PD) was estimated using Faith’s index based on the phylogenetic tree [34, 35]. The command *summary.single* was used to calculate the Shannon index in Mothur [30]. The *picante* package [36] was used to estimate the Faith’s PD [35] in R.

To determine the dissimilarity within bacterial community composition between any of the two sample pairs, we calculated the Bray-Curtis and unweighted UniFrac dissimilarity matrices [37]. Based on both of the matrices, non-metric multidimensional scaling (NMDS) was performed to estimate dissimilarities between water samples by using the *vega*n [38] and *picante* [36] packages in R. PERMANOVA (Adonis) was used to test the significance of patterns observed in NMDS. To demonstrate the relative abundance of the top ten genera in the different treatments, *HemI* software was used to generate the heatmap [39].

The function *cca* of *vegan* package was employed to explore the relationships between environmental variables and microbial community structure. The unimodal distribution pattern of bacterial communities along environmental gradient determined CCA (canonical correspondence analysis) rather than RDA (redundancy analysis) was the better method to explain the relationship. Environmental matrix and microbial community matrix were calculated by Euclidean distance and Bray-Curtis dissimilarity distance, respectively [40]. The Mantel test, run with 999 permutations, was employed to determine the relationships between the dissimilarities of bacterial communities and the Euclidean distance matrix of measured environmental variables [41]. Dissimilarities of bacterial community composition were calculated based on both the Bray-Curtis distance and unweighted UniFrac distance. The Mantel test was run using the *vegan* package in R [42].

### Nucleotide sequence accession numbers

The obtained raw data have been deposited in the NCBI short-reads archive database (Accession Number: SRP097674).

## Additional files

Additional file 1: **Figure S1.** Comparision of environmental factors under different temperatures. TOC, total organic carbon; DO, dissolved oxygen; ORP, oxidation-reduction potential; TN, total nitrogen; TP, total phosphorus; NH_4_^+^-N, ammonia nitrogen; NO_3_^−^-N, nitrate nitrogen; NO_2_^−^-N, nitrite nitrogen. C, without addition of *Microcystis*; L, low *Microcystis* biomass treatment; H, high *Microcystis* biomass treatment. The same lowercase letter represents there was no significant difference for the environmental factors under different temperatures. (TIF 4274 kb)

Additional file 2: **Table S1.** Relative abundance of the dominant bacterial phyla/subphyla in water samples of different treatment groups. (PDF 24 kb)

Additional file 3: **Figure S2.** Relative abundance of the six dominant bacterial phyla/subphyla in water samples of different treatment groups. (A) different *Microcystis* addition treatments; (B) different temperature treatments. C, without addition of *Microcystis.* L, low *Microcystis* biomass treatment; H, High *Microcystis* biomass treatment. Significant differences between different treatment groups was indicated by asterisk (Duncan’s multiple range test, * P < 0.05). (TIF 3878 kb)

Additional file 4: **Figure S3.** Relative abundance of the dominant bacterial phyla/subphyla in water samples of different treatment groups. (A) *Microcystis* addition treatments, (B) different temperature treatments. C, without addition of *Microcystis*; L, low *Microcystis* biomass treatment; H, high *Microcystis* biomass treatment. (TIF 2026 kb)

Additional file 5 **Figure S4.** Canonical correspondence analysis (CCA) indicating the relationships between environmental variables and microbial community composition. Environmental matrix and microbial community matrix were calculated using the Euclidean distance and Bray-Curtis dissimilarity distance, respectively. Environmental variables were indicated with black arrows. C, without addition of *Microcystis*; L, low *Microcystis* biomass treatment; H, high *Microcystis* biomass treatment. (TIF 893 kb)

Additional file 6: **Table S2.** Results of the forward selection and ordination of canonical correspondence analysis (CCA). (PDF 73 kb)

Additional file 7: Methods and results of the supplementary experiments. (PDF 83 kb)

Additional file 8: **Figure S5.** Diversity of the bacterial communities derived from the *Microcystis*-sterilized and *Microcystis*-unsterilized groups (paired t-test). U, *Microcystis-*unsterilized groups; S, *Microcystis-*sterilized groups. Shannon, Shannon-Wiener index; PD, phylogenetic diversity. (TIF 47 kb)

Additional file 9 **Figure S6.** Non-metric multidimensional scaling (NMDS) of the bacterial communities based on (A) unweighted UniFrac dissimilarity matrix; (B) Bray-Curtis dissimilarity matrix. U, *Microcystis-*unsterilized group; S, *Microcystis-*sterilized group. The values of *R*^*2*^ and *P* indicated the results of PERMANOVA. (TIF 1506 kb)

Additional file 10: **Figure S7.** Dissimilarity of bacterial communities within and between the *Microcystis*-sterilized (S) and *Microcystis*-unsterilized (U) groups. (A) based on unweighted UniFrac dissimilarity matrix; (B) based on Bray-Curtis dissimilarity matrix. U, *Microcystis-*unsterilized group; S, *Microcystis-*sterilized group. The same lowercase letter indicates no significant difference between groups (Duncan’s multiple range test). (TIF 62 kb)

## Data Availability

The obtained raw data have been added to the NCBI short-reads archive database (Accession Number: SRP097674). Other data generated during the study are included in this manuscript and the additional files.

## Abbreviations

DO: Dissolved oxygen
H: High *Microcystis* biomass treatment
L: Low *Microcystis* biomass treatment
NH_4_^+^-N: Ammonia nitrogen
NO_2_^—^N: Nitrite nitrogen. C, without addition of *Microcystis*
NO_3_^—^N: Nitrate nitrogen
ORP: Oxidation-reduction potential
TN: Total nitrogen
TOC: Total organic carbon
TP: Total phosphorus
