# Supplementary figures and images for: Variations of bacterial community during the decomposition of Microcystis under different temperatures and biomass
Source: BMC Microbiol. 2019 Sep 4;19:207. doi: 10.1186/s12866-019-1585-5 (PMC6727399; doi:10.1186/s12866-019-1585-5)

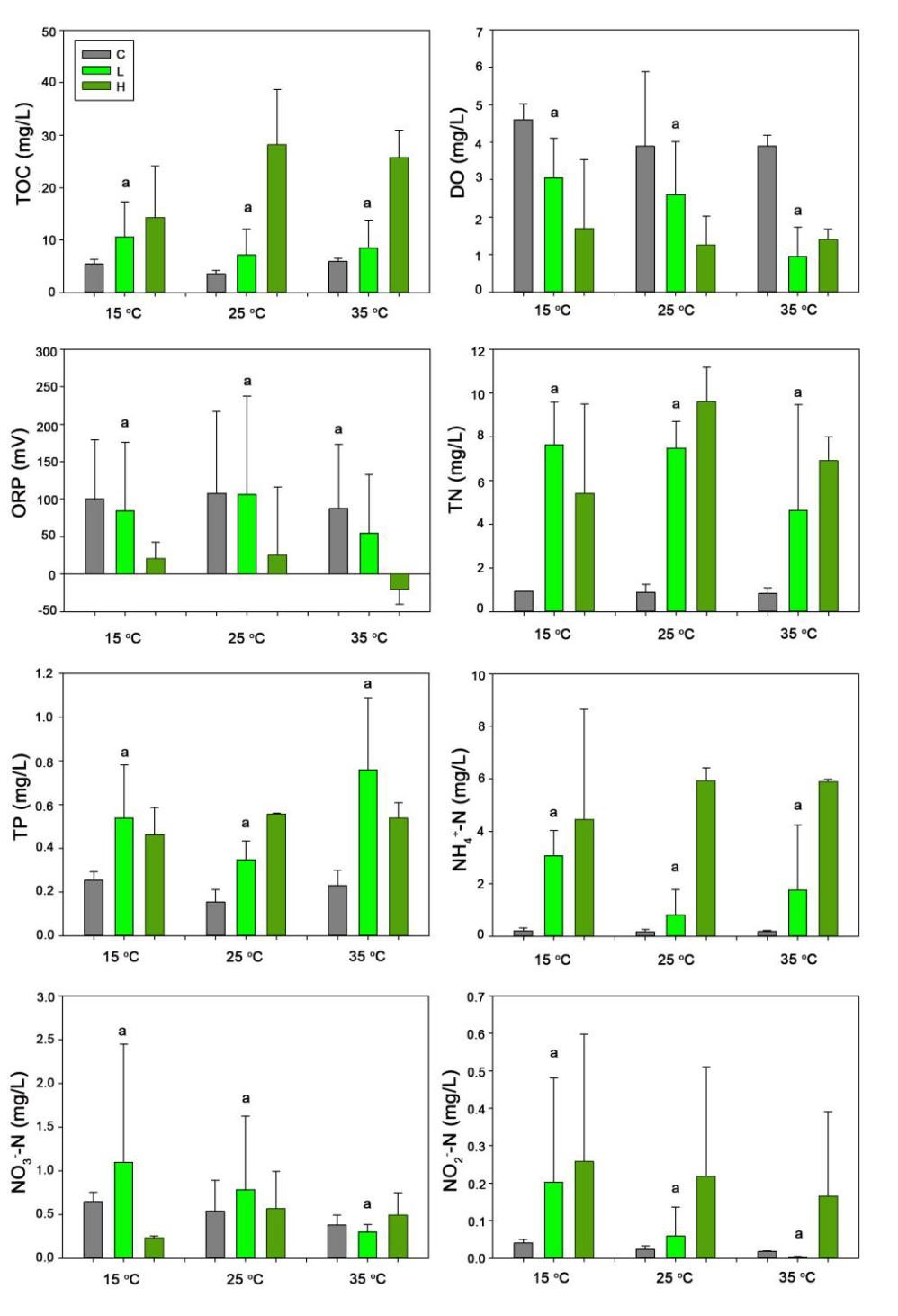

Supplement: Supplementary file 1 — Figure S1. Comparision of environmental factors under different temperatures. TOC, total organic carbon; DO, dissolved oxygen; ORP, oxidation-reduction potential; TN, total nitrogen; TP, total phosphorus; NH4+-N, ammonia nitrogen; NO3−-N, nitrate nitrogen; NO2−-N, nitrite nitrogen. C, without addition of Microcystis; L, low Microcystis biomass treatment; H, high Microcystis biomass treatment. The same lowercase letter represents there was no significant difference for the environmental factors under different temperatures. (TIF 4274 kb) [file 12866_2019_1585_MOESM1_ESM.tif]

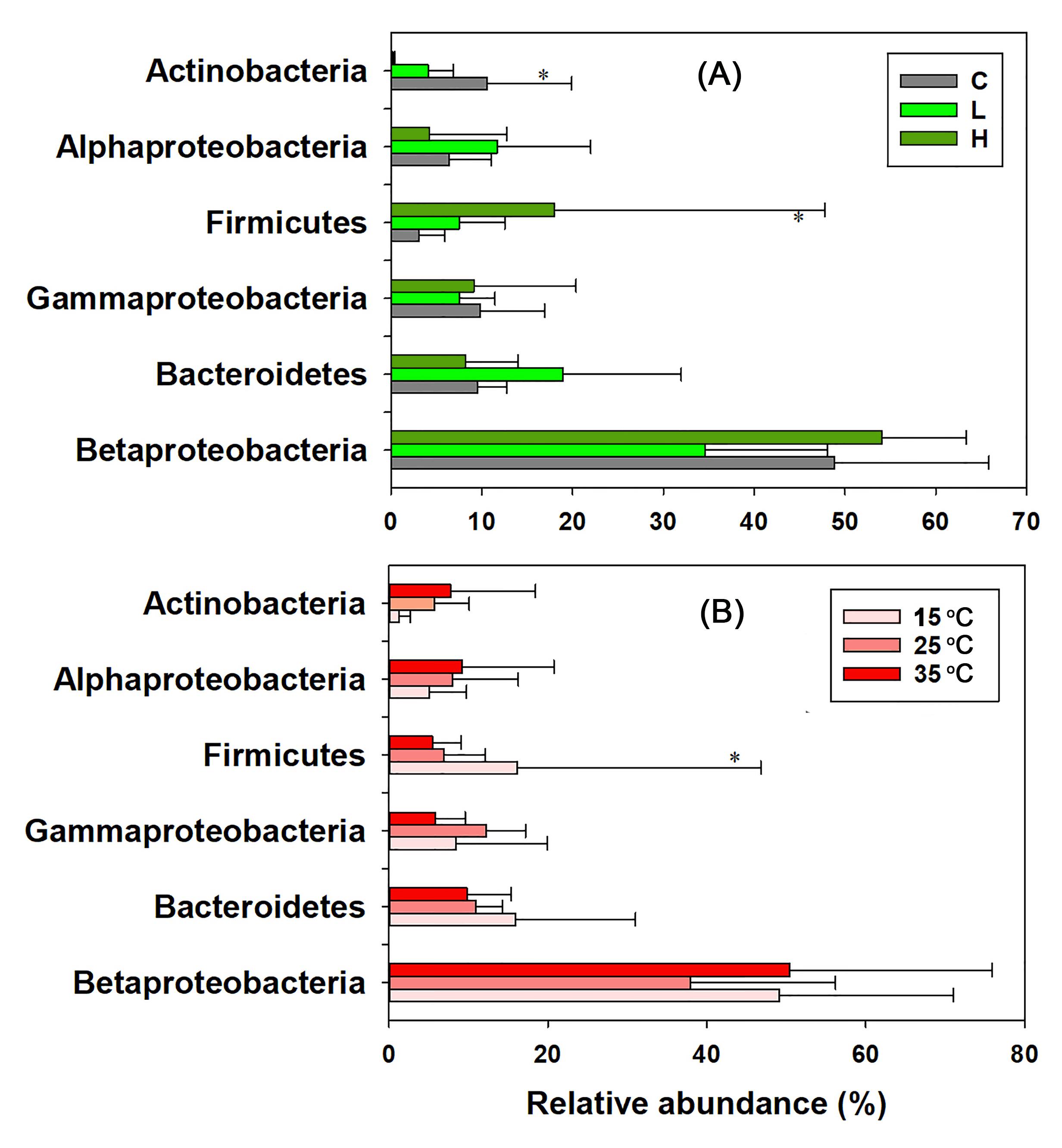

Supplement: Supplementary file 3 — Figure S2. Relative abundance of the six dominant bacterial phyla/subphyla in water samples of different treatment groups. (A) different Microcystis addition treatments; (B) different temperature treatments. C, without addition of Microcystis. L, low Microcystis biomass treatment; H, High Microcystis biomass treatment. Significant differences between different treatment groups was indicated by asterisk (Duncan’s multiple range test, * P < 0.05). (TIF 3878 kb) [file 12866_2019_1585_MOESM3_ESM.tif]

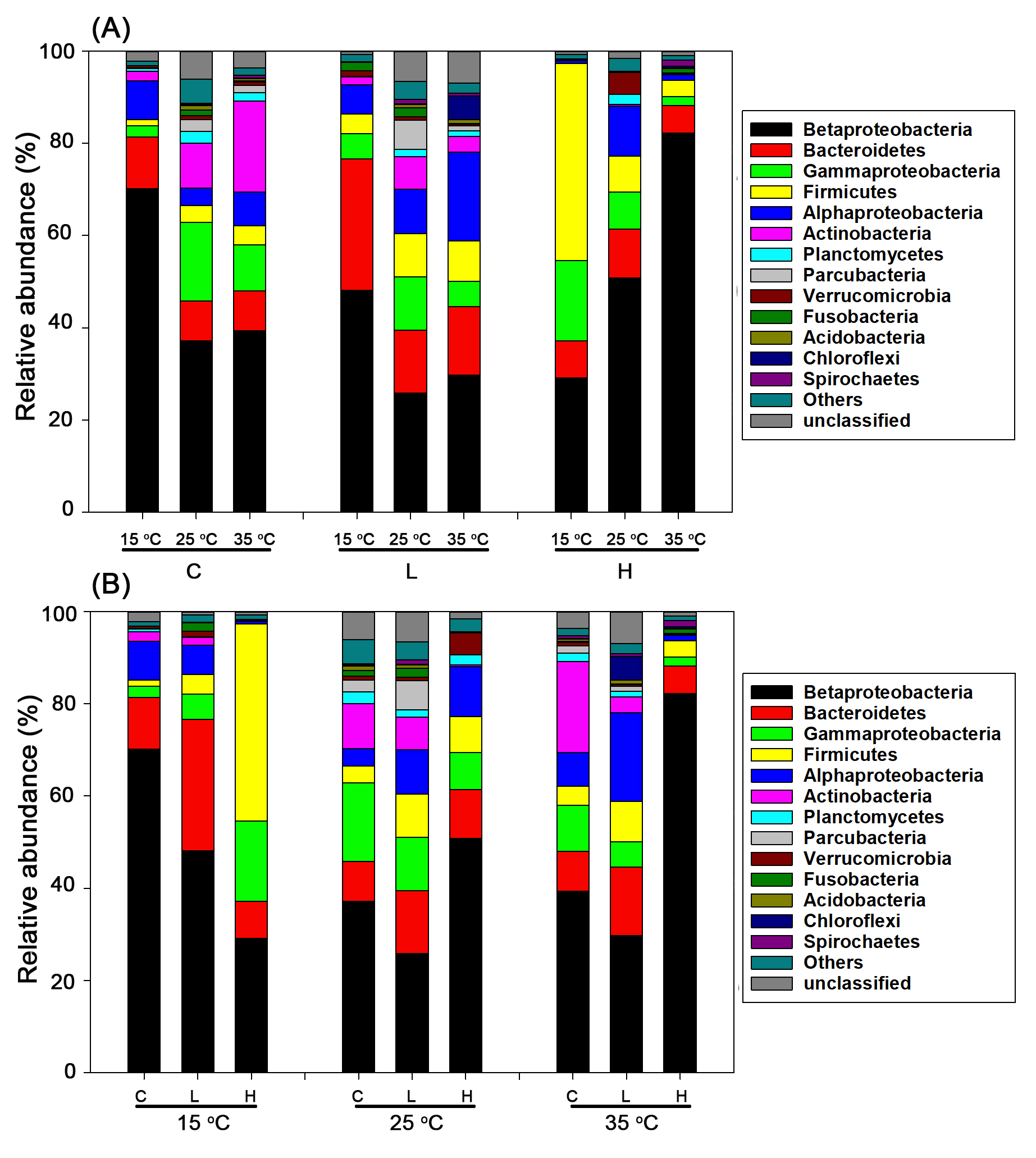

Supplement: Supplementary file 4 — Figure S3. Relative abundance of the dominant bacterial phyla/subphyla in water samples of different treatment groups. (A) Microcystis addition treatments, (B) different temperature treatments. C, without addition of Microcystis; L, low Microcystis biomass treatment; H, high Microcystis biomass treatment. (TIF 2026 kb) [file 12866_2019_1585_MOESM4_ESM.tif]

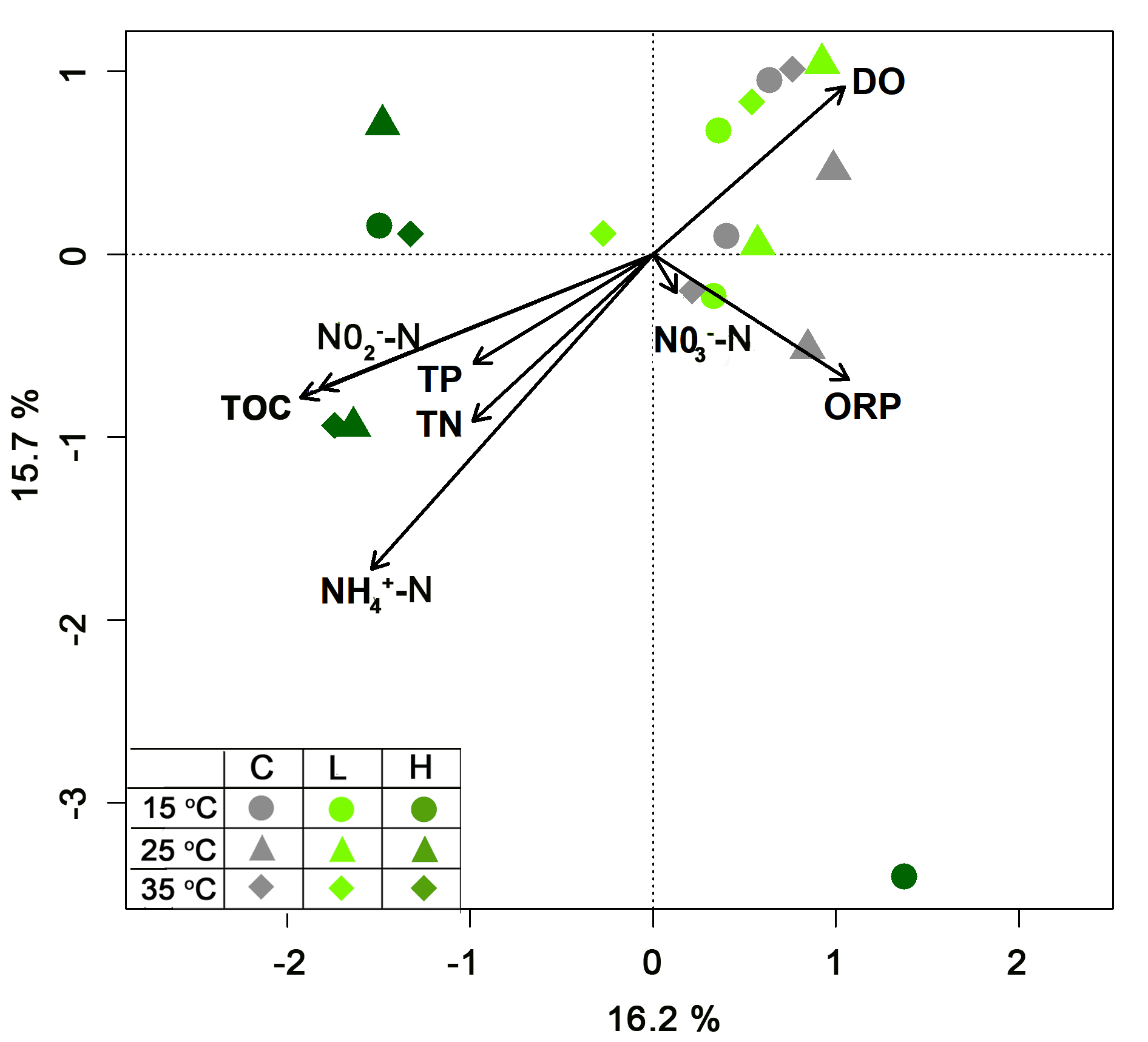

Supplement: Supplementary file 5 — Figure S4. Canonical correspondence analysis (CCA) indicating the relationships between environmental variables and microbial community composition. Environmental matrix and microbial community matrix were calculated using the Euclidean distance and Bray-Curtis dissimilarity distance, respectively. Environmental variables were indicated with black arrows. C, without addition of Microcystis; L, low Microcystis biomass treatment; H, high Microcystis biomass treatment. (TIF 893 kb) [file 12866_2019_1585_MOESM5_ESM.tif]

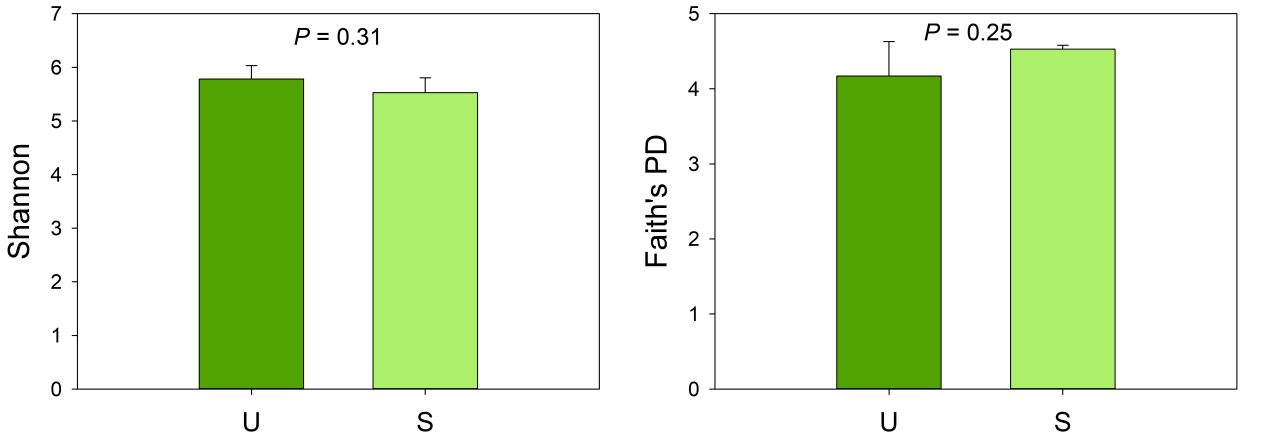

Supplement: Supplementary file 8 — Figure S5. Diversity of the bacterial communities derived from the Microcystis-sterilized and Microcystis-unsterilized groups (paired t-test). U, Microcystis-unsterilized groups; S, Microcystis-sterilized groups. Shannon, Shannon-Wiener index; PD, phylogenetic diversity. (TIF 47 kb) [file 12866_2019_1585_MOESM8_ESM.tif]

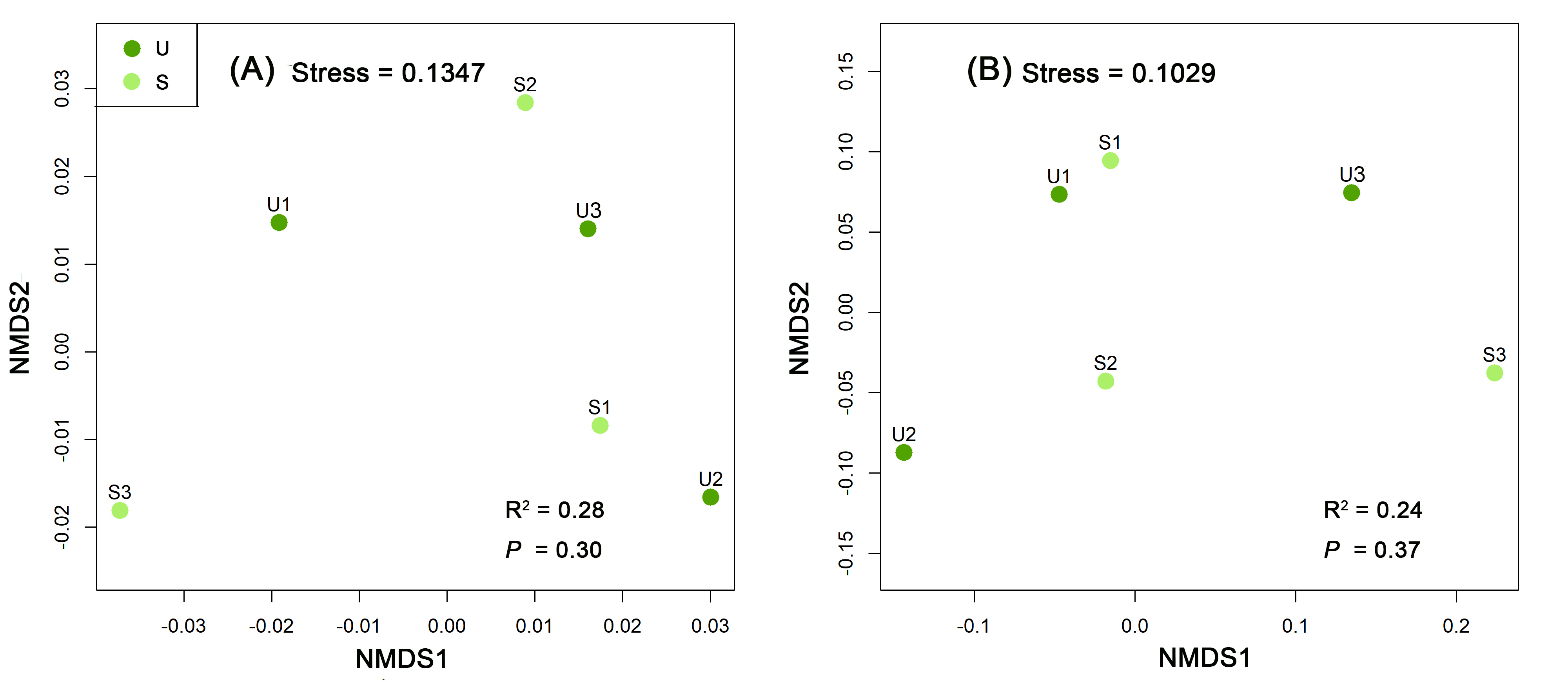

Supplement: Supplementary file 9 — Figure S6. Non-metric multidimensional scaling (NMDS) of the bacterial communities based on (A) unweighted UniFrac dissimilarity matrix; (B) Bray-Curtis dissimilarity matrix. U, Microcystis-unsterilized group; S, Microcystis-sterilized group. The values of R2 and P indicated the results of PERMANOVA. (TIF 1506 kb) [file 12866_2019_1585_MOESM9_ESM.tif]

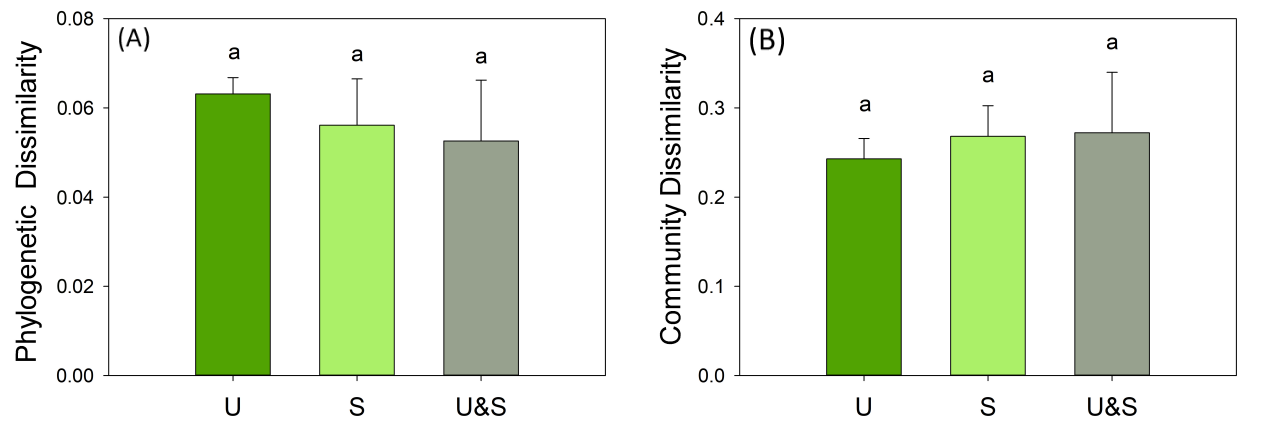

Supplement: Supplementary file 10 — Figure S7. Dissimilarity of bacterial communities within and between the Microcystis-sterilized (S) and Microcystis-unsterilized (U) groups. (A) based on unweighted UniFrac dissimilarity matrix; (B) based on Bray-Curtis dissimilarity matrix. U, Microcystis-unsterilized group; S, Microcystis-sterilized group. The same lowercase letter indicates no significant difference between groups (Duncan’s multiple range test). (TIF 62 kb) [file 12866_2019_1585_MOESM10_ESM.tif]
