# Supplementary material for: Variations of bacterial community during the decomposition of Microcystis under different temperatures and biomass
Source: BMC Microbiol. 2019 Sep 4;19:207. doi: 10.1186/s12866-019-1585-5 (PMC6727399; doi:10.1186/s12866-019-1585-5)
Supplement: Supplementary file 2 — Table S1. Relative abundance of the dominant bacterial phyla/subphyla in water samples of different treatment groups. (PDF 24 kb) [file 12866_2019_1585_MOESM2_ESM.pdf]

Table S1 Relative abundance of the dominant bacterial phyla/subphyla in water samples of different treatment groups.

Proportions (%)	15 ^{o}C			25 ^{o}C			35 ^{o}C		
	C	L	H	C	L	H	C	L	H
Betaproteobacteria	66.57	35.08	16.03	37.05	27.32	73.62	34.09	29.61	76.69
Gammaproteobacteria	2.40	2.08	3.52	21.24	10.29	8.65	11.19	5.21	2.75
Alphaproteobacteria	13.17	6.29	0.46	4.60	15.46	0.13	11.41	31.79	0.29
Bacteroidetes	7.73	45.04	1.44	7.52	15.51	7.39	11.61	17.67	10.28
Firmicutes	2.29	6.88	78.5	6.87	15.51	9.41	6.41	6.76	6.21
Hydrogenophaga	2.40	0.23	0	9.43	7.87	0.13	11.89	1.97	0.04
Planctomycetes	0.74	0.09	0	3.90	0.14	0	2.54	0.26	0.02
Parcubacteria	0	0	0	0.15	0.05	0	0.06	0.13	0
Verrucomicrobia	0.21	0.05	0	0.15	0.28	0.02	1.19	0.13	0
Fusobacteria	0	3.62	0	2.42	3.81	0.23	0.36	0.11	1.75
Acidobacteria	0.14	0.14	0	1.46	1.04	0	0.97	1.32	0.16
Chloroflexi	0	0	0	0	0.03	0	0.10	0.02	0
Spirochaetes	0	0	0	0.02	0.09	0	0	0	0.10
Hydrogenedentes	0	0	0	0.08	0.11	0	0	0.60	0
Gemmatimonadetes	0	0	0	0.06	0.02	0	0.26	0.09	0
Others	1.16	0.32	0	1.10	1.27	0.21	1.96	1.81	0.41
Unclassified	3.18	0.18	0.05	3.96	1.22	0.21	5.97	2.53	1.30

The relative abundance of bacterial phyla higher than 1% are dominant bacterial phyla. C, without addition of *Microcystis*; L, low *Microcystis* biomass; H, high *Microcystis* biomass.
