# Supplementary material for: Variations of bacterial community during the decomposition of Microcystis under different temperatures and biomass
Source: BMC Microbiol. 2019 Sep 4;19:207. doi: 10.1186/s12866-019-1585-5 (PMC6727399; doi:10.1186/s12866-019-1585-5)
Supplement: Supplementary file 6 — Table S2. Results of the forward selection and ordination of canonical correspondence analysis (CCA). (PDF 73 kb) [file 12866_2019_1585_MOESM6_ESM.pdf]

Table S2 Results of the forward selection and ordination of canonical correspondence analysis (CCA).

Physicochemical factors	CCA1	CCA2	R^2	*P*
ORP	0.985	0.174	0.811	0.001 ***
NH_4^+-N	-0.67	-0.742	0.503	0.006 **
TOC	-0.929	-0.371	0.41	0.028 *
NO_2^--N	-0.338	-0.941	0.274	0.093
DO	0.756	0.655	0.183	0.231
TN	-0.737	-0.676	0.17	0.246
TP	-0.858	-0.514	0.125	0.325
NO_3^--N	0.522	-0.853	0.006	0.962

ORP, oxidation-reduction potential; NH_4^+-N, ammonia nitrogen; TOC, total organic carbon; NO_2^--N, nitrite nitrogen; DO, dissolved oxygen; TN, total nitrogen; TP, total phosphorus; NO_3^--N, nitrate nitrogen. *$P < 0.05$, **$P < 0.01$, ***$P < 0.001$.
