# Supplementary material for: Variations of bacterial community during the decomposition of Microcystis under different temperatures and biomass
Source: BMC Microbiol. 2019 Sep 4;19:207. doi: 10.1186/s12866-019-1585-5 (PMC6727399; doi:10.1186/s12866-019-1585-5)
Supplement: Supplementary file 7 — Methods and results of the supplementary experiments. (PDF 83 kb) [file 12866_2019_1585_MOESM7_ESM.pdf]

Additional file 7

Methods and results of the supplementary experiments

Six microcosms were constructed according to the methods described in the manuscript. These microcosms could be divided into two groups. The *Microcystis*-sterilized group (S group) was added with sterilized *Microcystis* of 0.3 g/L. At the same time, the *Microcystis*-unsterilized group (U group) was added with unsterilized *Microcystis* of the same concentration. Each group has three replicates. The microcosms were incubated at 25 ^{o}C for 5 days. Water samples were collected and filtered. DNA was extracted and sequenced according to the methods described in the manuscript. The results of the supplementary experiment were shown as follows.

The richness and diversity of the bacterial community derived from the *Microcystis*-sterilized and *Microcystis*-unsterilized groups were shown in Fig. S5. By comparing the diversity indexes (Shannon-Wiener index and Faith's PD), comparable results were found between the bacterial communities derived from the two different treatment groups.

In order to compare the bacterial community composition derived from the two different treatment groups, the non-metric multidimensional scaling (NMDS) analysis was conducted. The pattern of NMDS indicated that sterilization of *Microcystis* would not significantly affect the composition of bacterial community in water of the microcosms (Fig. S6), which was also confirmed by the result of PERMANOVA.

Moreover, the unweighted UniFrac and Bray-Curtis distance within and between the U and S groups showed no significant difference (Fig. S7) ($P > 0.05$, Duncan's multiple range test).
